# Non-invasive Diagnosis of Antinephrin–Associated Podocytopathy

**DOI:** 10.1016/j.ekir.2025.05.005

**Published:** 2025-05-12

**Authors:** Felicitas E. Hengel, Michelle C.Z. Chong, Wing Yin Leung, Silke Dehde, Anne Mühlig, Matthias Janneck, Henry H.L. Wu, Tobias B. Huber, Arvind Ponnusamy, Nicola M. Tomas

**Affiliations:** 1 III. Department of Medicine, University Medical Center Hamburg-Eppendorf, Hamburg, Germany; 2 Hamburg Center for Kidney Health, University Medical Center Hamburg-Eppendorf, Hamburg, Germany; 3 Department of Renal Medicine, Lancashire Teaching Hospitals NHS Foundation Trust, Preston, UK; 4 Department of General Internal Medicine and Nephrology, Albertinen Hospital Hamburg, Hamburg, Germany; 5 Department of Pediatrics, University Medical Center Hamburg-Eppendorf, Hamburg, Germany; 6 Renal Research, Kolling Institute of Medical Research, Royal North Shore Hospital and The University of Sydney, Sydney, New Soulth Wales, Australia; 7 Faculty of Biology, Medicine and Health, The University of Manchester, Manchester, UK

**Keywords:** antinephrin autoantibodies, minimal change disease, podocytopathy, serology

## Abstract

**Introduction:**

Circulating autoantibodies against the podocyte surface protein nephrin have recently been described in patients with podocytopathies, that is, minimal change disease, primary focal segmental glomerulosclerosis, and childhood idiopathic nephrotic syndrome. Their high specificity for podocytopathies in combination with a strong correlation with disease activity hold the potential for a non-invasive diagnosis, but prospective data are lacking.

**Methods:**

Here, we describe 3 patients with contraindications or unwillingness for a kidney biopsy, hampering a timely histological diagnosis and choice of appropriate therapy.

**Results:**

In all patients, antinephrin autoantibodies were detected by quantitative immunoprecipitation, prompting the initiation of adequate treatment. These interventions induced a decrease in antinephrin autoantibody levels and clinical remission.

**Conclusion:**

Our study highlights the potential of antinephrin autoantibody measurement for a noninvasive diagnosis of antinephrin-associated podocytopathy.

## Graphical abstract

**Figure:**
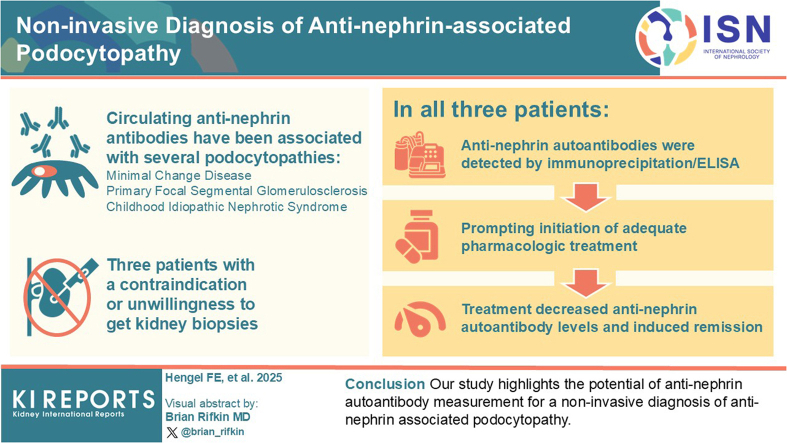

Minimal change disease and primary focal segmental glomerulosclerosis are immune-mediated primary podocytopathies usually presenting with severe nephrotic syndrome. Diagnostic biomarkers have long remained elusive; however, circulating autoantibodies against nephrin have recently been identified in patients with primary podocytopathies and were shown to strongly correlate with disease activity.^1^, ^2^, ^3^, ^4^ Importantly, patient-derived antinephrin positive IgG was demonstrated to induce proteinuria, nephrin phosphorylation, and a minimal change disease–like histotype upon transfer to a rabbit, thereby supporting a direct pathogenic role of these autoantibodies.^5^ These new insights into antinephrin-mediated pathomechanisms hold promise to improve our diagnostic and therapeutic approach toward affected patients.^6^^,^^7^ In this case series, we describe the diagnostic measurement of antinephrin autoantibodies in 2 patients with contraindications for a kidney biopsy and 1 patient refusing a kidney biopsy, allowing for a noninvasive diagnosis of antinephrin-associated podocytopathy and the corresponding treatment implications.

## Methods

Circulating antinephrin autoantibodies in patient serum samples were quantified using a hybrid technique of immunoprecipitation followed by enzyme-linked immunosorbent assay–based quantification of precipitated recombinant nephrin as described previously.^2^ Written informed consent for measurement and publication was obtained from all patients. Details of the methods are presented in the Supplementary Methods.

## Results

### Case 1

**Figure 1:**
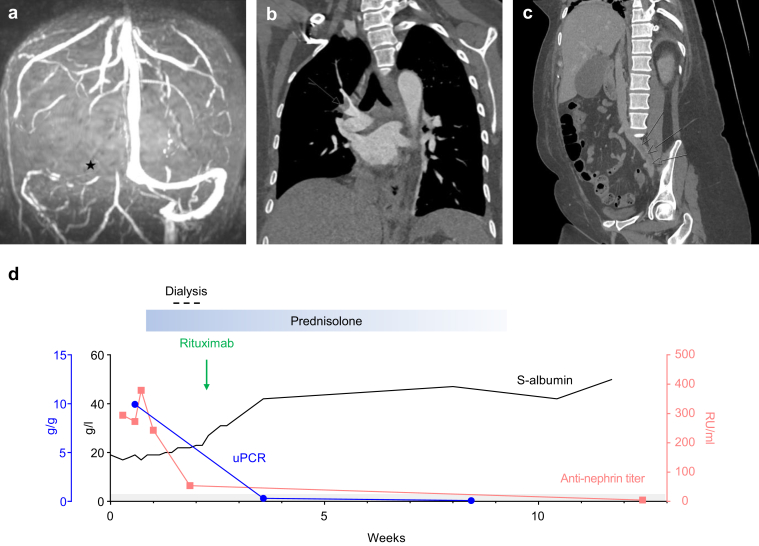
Antinephrin autoantibody measurement for noninvasive diagnosis of antinephrin-associated podocytopathy in a patient with severe nephrotic syndrome and thrombotic complications. (a) MR venogram showing no contrast flow in the right transverse sinus (asterisk) indicative of a right transverse sinus thrombosis. (b) CT thorax with contrast shows a filling defect in the segmental branch of the pulmonary artery to the right upper lobe (arrow). (c) CT abdomen and pelvis with contrast in the sagittal plane shows a nonocclusive filling defect through the length of the left common to the external iliac vein indicative of an iliac vein thrombosis (arrows). (d) Serum (S)-albumin levels (black, given as g/l), urinary protein-to-creatinine ratio (uPCR) (blue, given as g/g) and antinephrin autoantibody titers (red, given as relative units RU/ml) as determined by hybrid immunoprecipitation /ELISA; prednisolone treatment period (starting with 60 mg on day 6, rapid taper to 15 mg in week 7) is shown as a blue box, rituximab treatment period (day 16) is shown as a green arrow, and intermittent hemodialysis period (day 11 and day 15) shown as a dashed line. The grey area indicates the normal range of antinephrin antibody levels. CT, Computed tomography; ELISA, enzyme-linked immunosorbent assay; MR, Magnetic resonance.

A 49-year-old female patient presented to our institution with a 1-week history of worsening headache and lower limb edema. She received diuretic therapy and underwent a contrast-enhanced computed tomography scan of her brain, which showed cerebral venous sinus thrombosis (Figure 1a), necessitating anticoagulation therapy with low molecular weight heparin.

Within 48 hours, creatinine increased from 0.96 mg/ dl to 1.83 mg/dl (corresponding to 85 to 162 μmol/l), and further laboratory workup revealed a severe nephrotic syndrome with a serum albumin of 17 g/l, hyperlipidemia with cholesterol levels of 495 mg/dl (corresponding to 12.8 mmol/l), triglyceride levels of 700 mg/dl (corresponding to 7.9 mmol/l), and proteinuria with a urinary protein-to-creatinine ratio of 9.9 g/g (corresponding to 1124 mg/mmol). Urine tests did not show hematuria and serum immunological testing for possible causes of nephrotic syndrome gave negative results for anti-phospholipase A2 receptor (PLA2R) antibodies, lupus serology, serum electrophoresis, and free light chains. Hemodialysis was initiated because of rapidly progressive kidney function decline with fluid overload. A computed tomography scan of her chest, abdomen, and pelvis did not identify any malignancy but revealed pulmonary and iliac vein thromboembolisms (Figure 1b and c).

Because of the multiple and potentially life-threatening thromboembolic complications, anticoagulation could not be withdrawn for the performance of a kidney biopsy, preventing the histological diagnosis of the cause of the severe nephrotic syndrome. Considering this diagnostic dilemma, the patient was tested for antinephrin antibodies in an experimental setting, which revealed the diagnosis of antinephrin-associated podocytopathy (Figure 1d). The patient was commenced on oral prednisolone (60 mg daily) followed by 1 g of rituximab to deplete antinephrin autoantibodies and reduce prolonged use of high-dose corticosteroids. Three months after her initial presentation, antinephrin autoantibodies were undetectable and both kidney function and serum albumin had returned to the normal range (Figure 1d).

### Case 2

**Figure 2:**
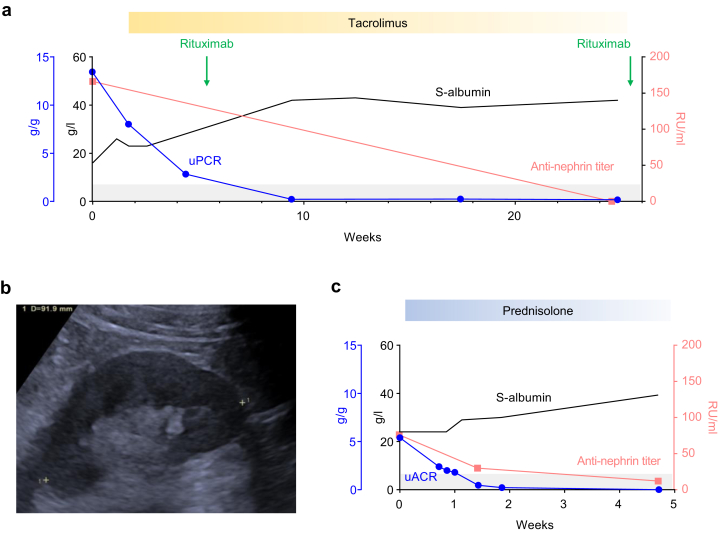
Antinephrin autoantibody measurement for noninvasive diagnosis of antinephrin-associated podocytopathy in a patient with complicated biopsy conditions and a patient with unwillingness for kidney biopsy. (a) Serum (S)-albumin levels (black, given as g/l) and urinary protein-to-creatinine ratio (uPCR) (blue, given in g/g) and antinephrin autoantibody titers (red, given as relative units RU/ml) as determined by hybrid immunoprecipitation enzyme-linked immunosorbent assay (ELISA); tacrolimus treatment period (starting with 2 mg twice daily in week 2, reduced to 1.5 mg, 1 mg, and 0.5 mg twice daily in week 10, 18, and 26, respectively) is shown as a yellow box and rituximab treatments (1 g as starting dose in week 6, 0.25 g as maintenance therapy in week 26) are shown as green arrows. (b) Renal ultrasound of the patient’s left kidney shows a reduced size in the patient with impaired procedural compliance. (c) S-albumin levels (black, given as g/l) and urinary albumin-to-creatinine ratio (uACR) (blue, given as g/g) and antinephrin autoantibody titers (red, given in relative units RU/ml) as determined by hybrid immunoprecipitation ELISA; prednisolone treatment period (starting with 70 mg daily in week 1, reduced to 20 mg daily in week 5) is shown as a blue box. The gray area indicates the normal range of antinephrin antibody levels.

A 49-year-old male patient with Down syndrome, obesity (body mass index > 40 kg/m^2^), metabolic liver disease, and chronic plaque psoriasis experienced an episode of ankle swelling and progressive dyspnea over 3 weeks. He presented to the emergency unit and was diagnosed with acute kidney injury and severe nephrotic syndrome; laboratory investigations revealed an increase in serum creatinine from a baseline of 0.77 mg/dl to 1.99 mg/dl (corresponding to 68 μmol/l and 176 μmol/l, respectively), a serum albumin of 16 g/l, hyperlipidemia with cholesterol levels of 388 mg/dl (corresponding to 10.03 mmol/l), and nephrotic range proteinuria with a urinary protein-to-creatinine ratio of 13.4 g/g (corresponding to 1514 mg/mmol) (Figure 2a). He was referred to the renal unit, where further workup, including renal ultrasound and immunology screen for possible causes of nephrotic syndrome as described above was unremarkable except for slightly reduced kidney size of 9 cm diameter (Figure 2b). He received i.v. diuretics and antiproteinuric therapy, including renin-angiotensin-aldosterone system inhibition, and anticoagulation agents. Steroid therapy was avoided because of the metabolic comorbidities of the patient. The patient was not able to tolerate the procedure of a kidney biopsy, again preventing the histological diagnosis of the severe nephrotic syndrome. The patient was instead tested for circulating antinephrin antibodies in an experimental setting, revealing the diagnosis of antinephrin-associated podocytopathy. Nonsteroidal immunosuppressive treatment with 2 mg tacrolimus twice daily was initiated and followed by 1 g rituximab, leading to full B-cell depletion and complete remission of nephrotic syndrome within 2 months (Figure 2a). Tacrolimus was slowly tapered to 0.5 mg twice daily. Clinical remission was retained 5 months after initial presentation with no detection of antinephrin autoantibodies (Figure 2a), and low-dose rituximab of 250 mg was planned for maintenance therapy, aiming at the subsequent full withdrawal of tacrolimus therapy.

### Case 3

A 48-year-old male patient presented with sudden-onset edema and weight gain of 8 kg, with no major medical history of comorbidities, infectious or allergic diseases. Laboratory investigations revealed a normal serum creatinine of 1.0 mg/dl (corresponding to 88 μmol/l), a serum albumin of 24 g/l, hyperlipidemia with a cholesterol level of 514 mg/dl (corresponding to 13.3 mmol/l), and nephrotic proteinuria with a urinary albumin-to-creatinine ratio of 5.4 g/g (corresponding to 610 mg/mmol) (Figure 2c). Urinary workup, renal ultrasound, and standard immunology screen for possible causes including anti-PLA2R antibodies were negative. The patient was unwilling to undergo a kidney biopsy, making a histological diagnosis of his nephrotic syndrome impossible. He was tested positive for circulating antinephrin antibodies, allowing for a diagnosis of antinephrin-associated podocytopathy, and started on oral steroids with prednisolone 70 mg daily as well as diuretics. In line with the relatively low antinephrin autoantibody titer at diagnosis, serum albumin rapidly increased and albuminuria decreased, which was paralleled by a drop in antinephrin autoantibody levels to the normal range (Figure 2c). His symptoms quickly disappeared and oral prednisolone was tapered within a few weeks.

## Discussion

Our case series illustrates the diagnostic potential of serological antinephrin antibody measurement in the context of nephrotic syndrome and contraindications to perform a kidney biopsy. Generally, a specific diagnosis of the disorder underlying nephrotic syndrome is of great importance because treatments greatly differ between etiologies. For example, patients with minimal change disease or primary focal segmental glomerulosclerosis require an instant start of steroids,^8^ most patients with anti-PLA2R-associated membranous nephropathy are monitored with an initial wait-and-see strategy potentially followed by immunosuppression,^8^ and underlying disease in secondary forms of nephrotic syndrome such as diabetic nephropathy^9^ or systemic lupus erythematosus requires distinct treatment of the specific primary disease.^10^

Usually, such specific diagnosis—as well as potential additional findings such as interstitial fibrosis, tubular injury, or a concomitant other disease—is revealed by a kidney biopsy, which is considered the diagnostic gold standard in the workup of nephrotic syndrome for most patients. However, kidney biopsies are sometimes contraindicated or associated with high risks in certain clinical settings, as illustrated by the first 2 cases of our report. Moreover, patients may refuse to undergo a kidney biopsy for personal reasons, like the third patient that we describe. Under such circumstances, a potential noninvasive diagnosis helps to appropriately initiate or withhold treatment, sparing the patient the adverse effects of, for example, high-dose glucocorticoid therapy. An excellent example in this direction is membranous nephropathy, where the current Kidney Disease: Improving Global Outcomes guidelines recommend to spare a kidney biopsy if anti-PLA2R autoantibodies are detected, but kidney function is normal.^8^ Based on the patients described herein and on the condition of prospective studies applying broadly available, clinically approved antinephrin antibody testing, we envision a similar strategy for patients with antinephrin-associated podocytopathies.

In summary, the detection of circulating antinephrin antibodies in patients with nephrotic syndrome can contribute valuable information on the specific disease etiology, enabling decision on appropriate treatment if a kidney biopsy is not possible or available. Larger and prospective studies as well as broadly available serological testing for the presence of antinephrin autoantibodies are urgently warranted.

## Disclosure

FEH, SD, TBH, and NMT report a pending patent in relation to the measurement of antinephrin antibodies. FEH reports honoraria from Novartis. TBH reports consulting fees from Boehringer Ingelheim, Novartis, Alexion, Pfizer, Retrophin-Travere, and Fresenius Medical Care. NMT reports consulting fees from Merida Bioscience and honoraria from CSL Vifor and AstraZeneca. All other authors declared no competing interests.

## Patient Consent

Written informed consent was obtained from all patients.

## Supplementary Material

Supplementary File (PDF) **Supplementary Methods.** STROBE Statement.

## Footnotes

Supplementary File (PDF)
Supplementary Methods. STROBE Statement.

## References

[1] Watts A.J.B., Keller K.H., Lerner G. (2022). Discovery of autoantibodies targeting nephrin in minimal change disease supports a novel autoimmune etiology. J Am Soc Nephrol.

[2] Hengel F.E., Dehde S., Lassé M. (2024). Autoantibodies targeting nephrin in podocytopathies. N Engl J Med.

[3] Raglianti V., Angelotti M.L., Cirillo L. (2024). Anti-slit diaphragm antibodies on kidney biopsy identify pediatric patients with steroid-resistant nephrotic syndrome responsive to second-line immunosuppressants. Kidney Int.

[4] Hengel F.E., Dehde S., Yilmaz A. (2025). Anti-nephrin autoantibodies in steroid-resistant nephrotic syndrome may inform treatment strategy. Kidney Int.

[5] Hengel F.E., Dehde S., Kretz O. (2025). Passive transfer of patient-derived anti-nephrin autoantibodies causes a podocytopathy with minimal change lesions. J Clin Invest.

[6] Cui Z., Zhao M.H. (2024). Anti-nephrin autoantibodies: a paradigm shift in podocytopathies. Nat Rev Nephrol.

[7] Reiser J., Ingelfinger J.R. (2024). Kidney disease and antinephrin antibodies. N Engl J Med.

[8] Kidney Disease: Improving Global Outcomes (KDIGO) Glomerular Diseases Work Group (2021). KDIGO 2021 clinical practice guideline for the management of glomerular diseases. Kidney Int.

[9] Rossing P., Caramori M.L., Chan J.C.N. (2022). Executive summary of the KDIGO 2022 clinical practice guideline for diabetes management in chronic kidney disease: an update based on rapidly emerging new evidence. Kidney Int.

[10] Rovin B.H., Ayoub I.M., Chan T.M. (2024). Executive summary of the KDIGO 2024 clinical practice guideline for the management of lupus Nephritis. Kidney Int.

