# Supplementary material for: Non-invasive Diagnosis of Antinephrin–Associated Podocytopathy
Source: Kidney Int Rep. 2025 May 12;10(8):2800–4. doi: 10.1016/j.ekir.2025.05.005 (PMC12347914; doi:10.1016/j.ekir.2025.05.005)
Supplement: Supplementary File (PDF) — Supplementary Methods. STROBE Statement. [file mmc1.pdf]

**Supplementary Material**

for

# Non-invasive diagnosis of anti-nephrin–associated podocytopathy

Felicitas E. Hengel[1,2*], Michelle C.Z. Chong[3*], Wing Yin Leung[3], Silke Dehde[1,2], Anne Mühlig[1,2,4], Matthias Janneck[5], Henry H.L. Wu[6], Tobias B. Huber[1,2,$], Arvind Ponnusamy[3,7,§], Nicola M. Tomas[1,2,§]

**Affiliations**

[1]III. Department of Medicine, University Medical Center Hamburg-Eppendorf, Hamburg, Germany

[2]Hamburg Center for Kidney Health, University Medical Center Hamburg-Eppendorf, Hamburg, Germany

[3]Department of Renal Medicine, Lancashire Teaching Hospitals NHS Foundation Trust, Preston, United Kingdom

[4]Immanuel Albertinen Krankenhaus Hamburg, Germany

[5]Department of Pediatrics, University Medical Center Hamburg-Eppendorf, Hamburg, Germany

[6]Renal Research, Kolling Institute of Medical Research, Royal North Shore Hospital & The University of Sydney, Sydney, Australia

[7]Faculty of Biology, Medicine & Health, The University of Manchester, Manchester, United Kingdom

**Table of Contents**

**Supplementary Methods**

PATIENT COHORT

GENERATION OF RECOMBINANT HUMAN NEPHRIN

QUANTIATIVE ANTI-NEPHRIN IP/ELISA ASSAY

STATISTICAL ANALYSIS

AUTHOR CONTRIBUTIONS

**STROBE Statement**

**Supplementary Methods**

PATIENT COHORT

Patients were cared for at the Department of Renal Medicine of the Lancashire Teaching Hospitals NHS Foundation Trust, Preston, United Kingdom (Case 1 and 2) and the Immanuel Albertinen Krankenhaus Hamburg, Germany (Case 3), between December 2023 and February 2025. Routine diagnostic procedures were conducted at corresponding local facilities. Individual follow-up periods were of observatory nature, depended on clinical disease course and are demonstrated in Figure 1 and 2.

Upon the diagnostic dilemma of severe nephrotic syndrome without the possibility for histological diagnosis, patients were informed about and agreed to the experimental test for anti-nephrin autoantibodies and serum samples were sent to the University Medical Center Hamburg-Eppendorf, Hamburg, Germany, for serological anti-nephrin antibody testing. Patients were selected for publication based on the following criteria: 1. Serological detection of anti-nephrin autoantibodies, 2. No kidney biopsy possible, 3. Follow-up available of at least 4 weeks, 4. Informed consent for publication.

GENERATION OF RECOMBINANT HUMAN NEPHRIN

The ectodomain of human nephrin (NCBI reference sequence: NM_004646.4, AA A25-G1037) in the eucaryotic expression vector pXLG was generously provided by Matthias Wilmanns (EMBL, Hamburg). The construct contained a N-terminal leader sequence for cellular secretion as well as a C-terminal Twin-Strep-tag and an 8x polyhistidine-tag for downstream purification and analysis. Sequence confirmation was achieved by Sanger sequencing and sequence alignment using Benchling (Biology Software, 2022-2023). The recombinant protein construct was expressed in human embryonic kidney (HEK) 293-6E cells as described before[2]. Seven days after transfection, cell culture supernatant was harvested, centrifuged, concentrated and the polyhistidine-tagged protein was purified under native conditions using NiNTA resin (Thermo Fisher Scientific) as described before. Protein quality and sample purity was validated by Western blot and/or Coomassie staining. The protein concentration was determined using a spectrophotometer (Biozym Scientific).

QUANTIATIVE ANTI-NEPHRIN IP/ELISA ASSAY

Anti-nephrin autoantibody titers were quantified in a two-step procedure of immunoprecipitation followed by quantification of immunoprecipitated recombinant nephrin as described before[2]. Firstly, 60 µl patient serum/plasma was mixed with 150 ng of recombinant human nephrin in RIPA buffer, incubated over night at constant rotation and exposed to MabCaptureC™ High Capacity Protein A Resin (Thermo Fisher Scientific) as described above. The resin was washed using Pierce™ Spin Columns (Thermo Fisher Scientific) twice in RIPA buffer followed by 4 washes in phosphate buffered saline (PBS) with 0.2% Tween 20 (PBS-T) and once in PBS. Protein elution was achieved by acidification using IgG elution buffer (Thermo Fisher Scientific) at pH 2.8 and neutralization with 1M Tris pH 9. Subsequently, immunoprecipitated recombinant Twin-Strep-tagged nephrin was quantified using a Streptactin-coated microplate (IBA Lifesciences GmbH). Wells were incubated with the eluate diluted in post coat buffer (TBS with BSA, Sigma-Aldrich) with 0.05% Tween 20 for 2 hours at 20 °C. Wells were washed four

times with TBS-T (Sigma-Aldrich) and incubated with anti-nephrin antibody (polyclonal sheep anti-nephrin antibody, R&D Systems; AF4269, diluted 1:1000 in post coat buffer with 0.05% Tween 20) overnight on a rocking platform at 4°C. Wells were washed four times and incubated with 100 µl of HRP-conjugated anti-shIgG (1:5,000, Jackson ImmunoResearch) for one hour at 20 °C. Wells were washed again before application of TMB ELISA peroxidase substrate solution (Avia Systems Biology) for 5 min at 20 °C, followed by acidification using 100 µl of 1 mol/L $H_3PO_4$-solution to stop the substrate reaction. The absorbance at 450 nm was determined using an ELISA reader (EL808, Bio-Tek instruments). The uper limit of the normal range was determined as follows: An anti-nephrin positive reference serum was titrated 1:2, 1:4, 1:8, 1:16, 1:32 and 1:64 and tested by immunoprecipitation/Western blot as described previously. The first dilution with a negative result was 1:32, which was set as the upper limit of the normal range in the immunoprecipitation/ELISA asssay. Consequently, this 1:32 dilution was included in every measurement as a cutoff. Quantification was done in duplicates and relative units (RU/ml) were determined in regard to a standard curve of serial dilutions of recombinant human nephrin.

STATISTICAL ANALYSIS

Not applicable.

AUTHOR CONTRIBUTIONS

TBH, AP and NMT conceptualized the study. NMT supervised the study. FEH, TBH, AP and NMT analysed the data. FEH, SD and AM performed experiments. MCZC, LW, MJ, HHLW and AP provided patient care. FEH and NMT wrote the manuscript with help from all authors.

STROBE Statement—checklist of items that should be included in reports of observational studies

| | Item No. | Recommendation | Page No. | Relevant text from manuscript |
|---|---|---|---|---|
| **Title and abstract** | 1 | (*a*) Indicate the study's design with a commonly used term in the title or the abstract | 1 | Anti-nephrin autoantibody measurement for a non-invasive diagnosis of anti-nephrin–associated podocytopathy |
| | | (*b*) Provide in the abstract an informative and balanced summary of what was done and what was found | 2 | Here, we describe three patients with contraindications or unwillingness for a kidney biopsy, hampering a timely histological diagnosis and choice of appropriate therapy. In all three patients, anti-nephrin autoantibodies were detected by quantitative immunoprecipitation/ELISA, prompting the initiation of adequate treatment. These interventions induced a decrease in anti-nephrin autoantibody levels and clinical remission. |
| **Introduction** | | | | |
| Background/rationale | 2 | Explain the scientific background and rationale for the investigation being reported | 2 | Minimal change disease (MCD) and primary focal segmental glomerulosclerosis (FSGS) are immune-mediated primary podocytopathies usually presenting with a severe nephrotic syndrome. Diagnostic |

| | | | | |
|---|---|---|---|---|
| | | | | biomarkers have long remained elusive; however, circulating autoantibodies against nephrin have recently been identified in patients with primary podocytopathies and were shown to strongly correlate with disease activity. Importantly, patient-derived anti-nephrin positive IgG was demonstrated to induce proteinuria, nephrin phosphorylation and an MCD-like histotype upon transfer to a rabbit, supporting a direct pathogenic role of these autoantibodies. These new insights into anti-nephrin–mediated pathomechanisms hold promise to improve our diagnostic and therapeutic approach towards affected patients. |
| Objectives | 3 | State specific objectives, including any prespecified hypotheses | 2 | N/A |
| **Methods** | | | | |
| Study design | 4 | Present key elements of study design early in the paper | 2 | In this case series, we describe the diagnostic measurement of anti-nephrin autoantibodies in two patients with contraindications for a kidney biopsy and one patient refusing a kidney biopsy, allowing for a |

| | | | | |
|---|---|---|---|---|
| | | | | non-invasive diagnosis of anti-nephrin–associated podocytopathy and the corresponding treatment implications. |
| Setting | 5 | Describe the setting, locations, and relevant dates, including periods of recruitment, exposure, follow-up, and data collection | Supplementary Material | See Supplementary Material |
| Participants | 6 | (*a*) *Cohort study*—Give the eligibility criteria, and the sources and methods of selection of participants. Describe methods of follow-up<br>*Case-control study*—Give the eligibility criteria, and the sources and methods of case ascertainment and control selection. Give the rationale for the choice of cases and controls<br>*Cross-sectional study*—Give the eligibility criteria, and the sources and methods of selection of participants | | N/A |
| | | (*b*) *Cohort study*—For matched studies, give matching criteria and number of exposed and unexposed<br>*Case-control study*—For matched studies, give matching criteria and the number of controls per case | | N/A |
| Variables | 7 | Clearly define all outcomes, exposures, predictors, potential confounders, and effect modifiers. Give diagnostic criteria, if applicable | | N/A |
| Data sources/ measurement | 8* | For each variable of interest, give sources of data and details of methods of assessment (measurement). Describe comparability of assessment methods if there is more than one group | | N/A |
| Bias | 9 | Describe any efforts to address potential sources of bias | | N/A |
| Study size | 10 | Explain how the study size was arrived at | Supplementary Material | See Supplementary Material |

Continued on next page

| | | | |
|---|---|---|---|
| Quantitative variables | 11 | Explain how quantitative variables were handled in the analyses. If applicable, describe which groupings were chosen and why | N/A |
| Statistical methods | 12 | (*a*) Describe all statistical methods, including those used to control for confounding | N/A |
| | | (*b*) Describe any methods used to examine subgroups and interactions | N/A |
| | | (*c*) Explain how missing data were addressed | N/A |
| | | (*d*) *Cohort study*—If applicable, explain how loss to follow-up was addressed<br>*Case-control study*—If applicable, explain how matching of cases and controls was addressed<br>*Cross-sectional study*—If applicable, describe analytical methods taking account of sampling strategy | N/A |
| | | (*e*) Describe any sensitivity analyses | N/A |
| **Results** | | | |
| Participants | 13* | (a) Report numbers of individuals at each stage of study—eg numbers potentially eligible, examined for eligibility, confirmed eligible, included in the study, completing follow-up, and analysed | N/A |
| | | (b) Give reasons for non-participation at each stage | N/A |
| | | (c) Consider use of a flow diagram | N/A |
| Descriptive data | 14* | (a) Give characteristics of study participants (eg demographic, clinical, social) and information on exposures and potential confounders | N/A |
| | | (b) Indicate number of participants with missing data for each variable of interest | N/A |
| | | (c) *Cohort study*—Summarise follow-up time (eg, average and total amount) | N/A |
| Outcome data | 15* | *Cohort study*—Report numbers of outcome events or summary measures over time | N/A |
| | | *Case-control study*—Report numbers in each exposure category, or summary measures of exposure | N/A |
| | | *Cross-sectional study*—Report numbers of outcome events or summary measures | N/A |
| Main results | 16 | (*a*) Give unadjusted estimates and, if applicable, confounder-adjusted estimates and their precision (eg, 95% confidence interval). Make clear which confounders were adjusted for and why they were included | N/A |
| | | (*b*) Report category boundaries when continuous variables were categorized | N/A |
| | | (*c*) If relevant, consider translating estimates of relative risk into absolute risk for a meaningful time period | N/A |

Continued on next page

| | | | | |
|---|---|---|---|---|
| Other analyses | 17 | Report other analyses done—eg analyses of subgroups and interactions, and sensitivity analyses | | N/A |
| **Discussion** | | | | |
| Key results | 18 | Summarise key results with reference to study objectives | 5 | In summary, the detection of circulating anti-nephrin antibodies in patients with nephrotic syndrome can contribute valuable information on the specific disease etiology, enabling decision on appropriate treatment if a kidney biopsy is not possible or available. |
| Limitations | 19 | Discuss limitations of the study, taking into account sources of potential bias or imprecision. Discuss both direction and magnitude of any potential bias | | N/A |
| Interpretation | 20 | Give a cautious overall interpretation of results considering objectives, limitations, multiplicity of analyses, results from similar studies, and other relevant evidence | 5 | In summary, the detection of circulating anti-nephrin antibodies in patients with nephrotic syndrome can contribute valuable information on the specific disease etiology, enabling decision on appropriate treatment if a kidney biopsy is not possible or available. Larger and prospective studies as well as broadly available serological testing for the presence of anti-nephrin autoantibodies are urgently warranted. |
| Generalisability | 21 | Discuss the generalisability (external validity) of the study results | | N/A |
| **Other information** | | | | |
| Funding | 22 | Give the source of funding and the role of the funders for the present study and, if applicable, for the original study on which the present article is based | 5 | see Acknowledgements |

*Give information separately for cases and controls in case-control studies and, if applicable, for exposed and unexposed groups in cohort and cross-sectional studies.

**Note:** An Explanation and Elaboration article discusses each checklist item and gives methodological background and published examples of transparent reporting. The STROBE checklist is best used in conjunction with this article (freely available on the Web sites of PLoS Medicine at http://www.plosmedicine.org/, Annals of Internal Medicine at http://www.annals.org/, and Epidemiology at http://www.epidem.com/). Information on the STROBE Initiative is available at www.strobe-statement.org.
